# Supplementary material for: Impact of Endophthalmitis on the Risk of Acute Myocardial Infarction in Ankylosing Spondylitis Patients: A Population-Based Retrospective Cohort Study
Source: J Clin Med. 2023 Feb 3;12(3):1211. doi: 10.3390/jcm12031211 (PMC9918242; doi:10.3390/jcm12031211)
Supplement: Supplementary file 1 [file jcm-12-01211-s001.zip › jcm-2109162-supplementary.pdf]

Supplementary Materials

Table S1. The ICD-9-CM codes, and definitions used in this study for data extraction and analysis.

Study Population:	ICD-9-CM Codes/Definition
Ankylosing spondylitis (AS)	720.0; Outpatient visits≧ 3 or inpatient
Endophthalmitis	360.0, 360.00-360.04, 360.1
**Events:**	
Acute myocardial infarction (AMI)	410
**Comorbidities:**	Baseline: 1 year before inclusion date and medical visits ≧3; Endpoint: 1 year before the incidence of AMI, the date of withdrawal from the insurance system, or the tracking endpoint and medical visits ≧3
Diabetes mellitus (DM)	250
Hyperlipidemia	272
Hypertension (HTN)	401–405
Cerebrovascular accident (CVA)	430–438
Congestive heart failure (CHF)	428
Chronic obstructive pulmonary disease (COPD)	490-492, 494, 496
Asthma	493
Coronary artery disease (CAD)	413–414
Charlson comorbidity index revised (CCI_R)	CCI removed AMI, DM, HTN, CVA, CHF, COPD, Asthma, and CAD

Table S2. Years of follow-up.

Endophthalmitis	Min	Median	Max	Mean ± SD	*p*
Total	0.01	7.80	15.99	9.91 ± 8.57	0.762
With	0.01	7.71	15.99	9.86 ± 8.43	
Without	0.01	7.82	15.99	9.92 ± 8.58	

p: Mann-Whitney U-test

Table S3. Years to AMI.

Endophthalmitis	Min	Median	Max	Mean ± SD	*p*
Total	0.03	2.09	15.88	3.50 ± 4.08	<0.001
With	0.03	1.92	15.82	2.91 ± 3.17	
Without	0.03	2.24	15.88	3.64 ± 4.19	

p: Mann-Whitney U-test
